# *Lactococcus lactis* : A New Strategy for Vaccination

**Published:** 2017

**Authors:** Maryam Azizpour, Seyyed Davood Hosseini, Parvaneh Jafari, Neda Akbary

**Affiliations:** 1- Department of Microbiology, Arak branch, Islamic Azad University, Arak, Iran; 2- Razi Vaccine and Serum Research Institute, Arak Branch, Arak, Iran; 3- Department of Microbiology, Islamic Azad University, Arak Branch, Arak, Iran

**Keywords:** DNA, *Lactococcus lactis*, Vaccines

## Abstract

Needle free vaccines have a several advantages and very attractive way for vaccination. In a body, mucosal surfaces provide a universal entry portal for all the known and emerging infectious pathogenic microbes. Therefore, it seems, vaccination strategies need to be reorganized for vaccines that are hindering the entry capability of pathogenic microbes through mucosal surfaces. Lactic acid Bacteria (LAB) are widely used in the food industry and at the present, used as delivery vehicles for biological investigations. In this review, we summarized the Results of several studies which *Lactococcus lactis* (*L. lactis*) used as a live vector for vaccines. These bacteria are considered as promising candidates for heterologous expression of proteins and biotechnological usage. LAB are considered as promising candidates for heterologous expression of proteins and biotechnological usage. The results showed that these bacteria have an ability to deliver antigen to immune system. Therefore, developing mucosal live vaccines using lactic acid bacterium, *L. lactis*, as an antigen delivery vector, is an attractive alternative choice and a safer vaccination strategy against pathogens.

## Introduction

In 1980, Walter Schaffner demonstrated that the bacteria are able to transfer genetic material into mammalian cells *in vitro*. So, they suggested new vectors for plasmid vaccines transfer ^1–3^. Later, it was shown that the gram-positive bacteria like *Listeria monocytogenes* are capable of conveying DNA plasmid ^4^. Since then, attenuated or artificially engineered invasive bacteria have been tested as a vehicle for transgene delivery ^5^.

For centuries, people have recognized that the consumption of fermented products can have a positive effect on human health. Over decades, it has become clear that these probiotic, Lactic Acid Bacteria (LAB) are classified as safe GRAS by the United States Food and Drug Administration (USFDA) ^6^. Moreover, a number of LAB can induce the immune system response like adjuvants, because of their probiotic properties and their capacity for inducing the host immune system ^7^. While commensal and pathogenic bacteria as a mucosal delivery vehicles have benefits and drawbacks, lactic acid bacteria are more desirable for their safety and lower side effects ^8^.

*Lactococcus lactis (L. lactis)* with a good history of safety in food fermentation and the ability to survive in passage through the gastrointestinal tract of animals and humans ^9^ (until now, with a 2 to 3 days survival time) does not invade or colonize the mucosal surfaces of the host. Furthermore, *L. lactis* does not have lipopolysaccharides and for this reason, does not stimulate host immune responses powerfully ^10–12^. Because of the progress in many genetic tools and sequenced complete genome, it is easier for researchers to manipulate the gene and produce proteins to the host mucosal surfaces, *via* the oral, genital or intranasal ^12–15^. Now, many studies are designed which use recombinant *L. lactis* to stimulate an immune response against various antigens ^9^.

In this paper, the ability of *L. lacti*s to transfer antigenic and therapeutic proteins was described. For this purpose, first, the interaction between *L. lacti*s and host gastrointestinal mucosal tract was explained. So, new investigations which use the recombinant *L. lacti*s as a mucosal vaccine were reviewed. Eventually, some early outcomes of such antigen producing bacteria were included in this study in order to pave the way for future developments.

### *L.* lactis and host interaction

**Figure 1.**
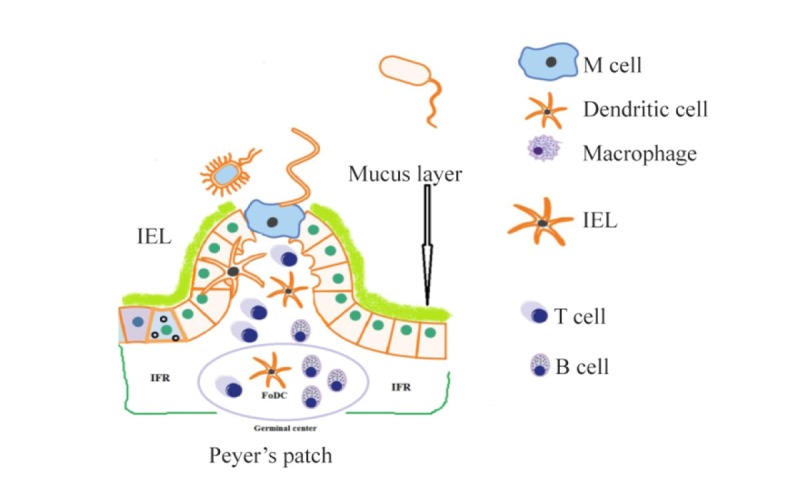
Schematic representation of Peyer’s patches, M cells, and the different immune cell populations. M cells have no mucus. IFR: intra-follicular region, B: B cells, IEL: intra-epithelial lymphocyte, T: T cells, FoDC: fol-licular dendritic cell, DC: dendritic cells.

Microfold (M) cells have a significant role in inducing mucosal immune response and perpetuity of the mucosal surface barrier. M cells transfer pathogens and foreign molecules from apical lumen side to basal side *via* using transcytosis. M cells do not have a mucus layer on their apical side ^5,16^. This character allows M cells to uptake antigens efficiently from the luminal space. The basal side of M cells, which formed from invaginated membranes, has pockets and house Dendritic Cells (DCs) (Figure 1). These DCs take up transported pathogens and molecules and help to manage the adaptive immune response ^17^. This close vicinity of DCs to M cells is especially remarkable because of the rapid process of the transcytosed antigens and presentation of antigenic peptides to B and T cells for inducing immune responses. Germinal center contains a net-work of follicular dendritic cells and many B cells, IgA-producing B cells ^16^. These B cells can migrate into the intestinal lamina propria and secrete IgA (sIgA, Figure 1). The space between neighborhood follicles in the Peyer’s Patches (PPs) is called Intrafollicular Region (IFR). The IFR is full of T cells and DCs and helps to administer the adaptive immune response in the PPs ^18^. *L. lactis* enters through Intestinal Epithelial Cells (IECs) or M cells, so internalizes and reproduces within phagocytic cells, and causes cellular death mechanism used to spread to a deeper layer. In a usual manner, inflammatory response induced and infiltration of polymorphonuclear cells occurred cause the activation of inflammatory cascades and produce pro-inflammatory cytokines and severe tissue damages. So, the microbes from infected lesions were cleared and the production of antimicrobial neutralizing antibodies occurred. Thus, a dynamic immune network with native and acquired mucosal responses was created ^19–21^{Adachi, 2010 #14}.

### *L.* lactis as a live vehicle for mucosal vaccine delivery

Developing the molecular ways and genetic manipulating to effectively produce antigens and curative molecules in various cells to deliver protein and DNA to host cells was important to present LAB as a live vehicle. A remarkable property of genetically-engineered LAB is that mucosal administration elicits both systemic and mucosal immunity ^12^. In LAB, a hopeful candidate for vaccines development is *L. lactis* because (1) various genetic ways have been developed for it, (2) its genome is completely sequenced, (3) and its safety property has been revealed. Iwaki *et al* in 1990 attempted to use *L. lactis* as a live vaccine ^22^. Many investigations with recombinant *L. lactis* strains have been performed and protection or incomplete protection was observed ^23^. Lately, LAB as a live vehicle has been investigated in different studies ^24–26^. In this study, some recent studies for using LAB as a vaccine are included.

## Results

The first investigation for *L. lactis* based mucosal vaccine was against the *Streptococcus mutans* surface protein (Pac). When cytoplasm expressed this gene in *L. lactis* and supplied orally the killed bacteria, the valuable responses of IgA and IgG were seen ^22^. In addition, next studies on *Clostridium tetani* toxin, fragment C (TTFC-Tetanus Toxin Fragment C) with *L. lactis* strain showed the highly immunogenic property ^6^,^27^. Studies showed that the nasal route of surface which displayed recombinant TTFC was preferred ^28^. The intracellularly expressed T3SS (type III secretory system protein) vaccines against EspB which were orally used, after ten days, have no particular serum and faucal antibodies. Besides, in BALB/c mice, intra-peritoneal vaccination of the EspB protein increases serum IgG and faucal IgA levels ^29^. The comparative efficacy was explored when given orally and intramuscularly in piglets ^30^. The intramuscular inoculation with recombinant *L. lactis* producing FaeG (fimbria adhesion) can stimulate a specific systemic response. In another study, nasal inoculation with recombinant *L. lactis* expressing a conserved stretch peptide of the avian influenza M2 antigen in birds can increase survival times against high pathogenic avian influenza virus A subtype H5N2 ^31^.

In another challenge on mice, nasal and Broncho-alveolar Lavages (BAL) inoculation with recombinant *L. lactis* expressing *Brucella abortus* (*B. abortus*) Cu-Zn Superoxide Dismutase (SOD), showed SOD-specific IgM and SOD-specific sIgA antibodies which protected the mice against virulent *B. abortus* strain ^9^. Oral and intra-nasal vaccination with *L. lactis strain* expressing *Rhodococcus equi (R. equi)* VapA (virulence-associated protein A) in mice led to a specific mucosal immune response against VapA in a challenge with a virulent strain of *R. equi* ^32^. In another investigation, intragastric route vaccination with recombinant *L. lactis* producing VP7 could induce systemic IgG antibody response against rotavirus ^33^. So, mice orally administered with recombinant *L. lactis* producing intracellular rotavirus spike-protein subunit VP8, showed the significant levels of intestinal IgA antibodies, while the secreted cytoplasm expressed protein or as a surface-anchored antigen induced anti-VP8 antibodies at both mucosal and systemic levels ^34^. Oral administration of recombinant *L. lactis* producing enterotoxin B of *Staphylococcus aureus (S. aureus)* in mice elicited cellular or systemic immune responses and increased survival rate in vaccinated mice against *S. aureus* ^14^. Moreover, vaccination of animal with *L. lactis* expressed papillomavirus type16 (HPV16) E7 protein, persuasion of humoral and cellular immune responses and rotected the animals against HPV-16 induced tumors ^34^. In mice, intranasal administration of recombinant *L. lactis* strain expressing *Yersinia pseudotuberculosis* Low-calcium response V (LcrV) antigen was able to elicit specific systemic and mucosal antibody and cellular immune responses against Yersinia infection. This investigation revealed that the type of antigen and administration place of vaccine are very important which can have an effect on antigen-specific immune responses ^35^,^36^. These studies are very valuable for the probability in applying vaccination or therapy with recombinant *L. lactis* because of their capacity for inducing mucosal and systemic immune responses ^37^,^38^.

### Few general strains of L. lactis and plasmids

NZ9000 is the usual standard host strain for nisin regulated gene expression (NICE®). Moreover, in this bacteria, nisK and nisR genes were cloned into the pepN gene of MG1363 ^39^. In the strain NZ9100, nisin genes were inserted into a neutral locus. All used strains were obtained from *L. lactis* subsp. *cremoris* MG1363.

In pNZ8008, pNZ8148, pNZ8149, and pNZ8150 vectors, replicon was the same and arose from pSH71 plasmid of *L. lactis*. These plasmids can be multiplied in various gram-positive bacteria, for example, *Streptococcus thermophilus* and *Lactobacillus plantarum (L. plantarum)* and they replicate in *Escherichia coli (E. coli)*, but need a recA+strain like MC1061. The pNZ8149 vector contains the lacF gene as a food grade selection marker. In such vectors for transformation process, a host strain, such a *L. lactis* NZ3900, which has lactose operon and lacks lacF gene, was necessary ^40^,^41^. In pNZ9530, the replication genes came from *Enterococcus faecalis* pAMß1 plasmid which replicate only in gram-positive bacteria, like, *L. lactis* and *L. plantarum* ^42^,^43^. In table 1, common host strains and plasmids are summarized.

**Table 1.** *L. lactis* strains and plasmids for expression

**Strains**	**Strains property**	**Plasmids**	**Plasmids property**	**Reference**
***L. lactis* NZ9000/NZ9100**	Refer text	pNZ8008	Reference plasmid for nisin, intracellular expression	[^42^, ^47^]
***L. lactis* NZ9000/NZ9100**	Refer text	pNZ8148	Cm^R,^ intracellular expression	[^42^]
***L. lactis* NZ9000/NZ9100**	Refer text	pNZ8150	Cm^R,^ intracellular expression	[^42^]
***L. lactis* NZ9000/NZ9100**	Refer text	pNZ9530	low copy plasmid, intracellular expression	[^42^, ^46^]
***L. lactis* NZ3000**	acF^−^ of strain MG5267	pNZ8149	lacF^+,^ food grade, intracellular expression	[^44^, ^48^]
***L. lactis* NZ3900**	lacF-, pepN: nisRK, food grade	pNZ8149	lacF^+,^ food grade, intracellular expression	[^44^, ^48^]
***L. lactis* NZ3910**	Same as but nisRnisK integrated into a neutral locus	pNZ8149	lacF^+,^ food grade, intracellular expression	[^49^, ^48^]
***L. lactis* NZ9000/NZ9100**	Refer text	pNZ8120	Cm^R,^ NICE Secretion vectors	[^50^]
***L. lactis* NZ9000/NZ9100**	Refer text	pNZ8121	Cm^R,^ NICE Secretion vectors	[^50^], unpublished
***L. lactis* NZ9000/NZ9100**	Refer text	pNZ8122	Cm^R,^ NICE Secretion vectors	[^51^]
***L. lactis* NZ9000/NZ9100**	Refer text	pNZ8123	Cm^R,^ NICE Secretion vectors	unpublished
***L. lactis* NZ9000/NZ9100**	Refer text	pNZ8124	Cm^R,^ NICE Secretion vectors	[^52^], unpublished
***L. lactis* NZ3900/NZ3910**	Refer Table 1	pNZ8151	lacF^+,^ food grade, intracellular expression	[^42^]
***L. lactis* NZ9130**	alr-, nisRK	pNZ8152	lacF^+,^ food grade, intracellular expression	[^49^, ^42^]

Cm^R^: Chloramphenicol resistance.

### Safety concerns

The potential risk of using lactic acid bacteria based mucosal vaccines is the entry of the genetically manipulated creatures to the environment. The manipulated bacteria which produce antigens and antibiotic markers may lead to the horizontal transfer of plasmid to other bacteria. Therefore, the auxotrophic mutants which are unable to multiply in the environment were designed. For this reason, in *L. lactis*, scientists substituted the thyA gene (thymidylate synthase) with the human IL–10 and made an auxotrophic strain which could not survive in an environment without thymidine ^44^. So, a recombinant *L. lactis* was made which contained LLO (Listeriolysin O of *Listeria monocytogenes*) gene. Therefore, such bacteria not only need a vector with antibiotic markers but also minimize the probability of gene transfer to another bacteria in the environment ^45^. Also, a novel vaccination method was the external linkage of ARV (avian retro virus) sigma C to LAB cell wall. When this antigen was cloned in *E. coli* and conjugated on the surface of *Enterococcus faecium*, it induced mucosal and systemic immunity in mouse ^46^.

## Conclusion

A big concern about the use of live LAB mucosal vaccines was the risk of transmission of genetically manipulated creatures to nature. So, the use of auxotrophic mutants can prevent the reproduction of such organisms in the environment. Also, food grade plasmids and auxotrophic strains can be used for solving the problem about the horizontal transfer of plasmids which carry antibiotic resistance markers to the environmental and host microflora.

In this paper, some LAB mucosal vaccines were reviewed which had some advantages in comparison to injected vaccines: (a) their ability to induce the systemic and mucosal immune responses in the host cell, (b) their easy manipulation (c) not requiring expert personnel. Moreover, its safety concerns about releasing recombinant plasmids and chromosomally modified bacterial strains in the environment can be controlled. So, lactic acid bacteria are very good mucosal delivery vectors for heterologous antigens and can be used in clinical trials. The studies revealed that recombinant *L. lactis* can stimulate mucosal immunity response. So, vaccination or therapy strategy with these bacteria is valuable.
